# Supplementary material for: Label-Free Fluorescence Molecular Beacon Probes Based on G-Triplex DNA and Thioflavin T for Protein Detection
Source: Molecules. 2021 May 17;26(10):2962. doi: 10.3390/molecules26102962 (PMC8156537; doi:10.3390/molecules26102962)

Supporting Information

Label-free Fluorescence Molecular Beacon Probes Based on G-Triplex DNA and Thioflavin T for Protein Detection

Jun Xue, Jintao Yi*, and Hui Zhou*

College of Chemistry and Chemical Engineering, Gannan Normal University, Ganzhou 341000, China

E-mail: yijt@hnu.edu.cn; huizhou314@163.com

Table S1. Sequences of DNA probes used in this work.

Name	Sequence (5'-3')
G3MB0	TGGGTAGGGCGGGTTTAAGCCTTTTTTTGGCTTAAA
G3MB1	TGGGTAGGGCGGGTTTAATTTTTTTTTAAACCC
G3MB3	TGGGTAGGGCGGGTTTAATTTTTTTTTAAACCCGCCC
G3MB4	TGGGTAGGGCGGGTTTAATTTTTTTTTAAACCCGCCCTACCC
G4MB0	AGGG TTAGGGTTAGGGTTAGGGTTTAAGCCTTTTTTTGGCTTAAA
G4MB1	AGGG TTAGGGTTAGGGTTAGGGTTTAATTTTTTTTTAAACCC
G4MB2	AGGG TTAGGGTTAGGGTTAGGGTTTAATTTTTTTTTAAACCCTAA
G4MB3	AGGG TTAGGGTTAGGGTTAGGGTTTAATTTTTTTTTAAACCCTAACCC
G4MB4	AGGG TTAGGGTTAGGGTTAGGGTTTAATTTTTTTTTAAACCCTAACCCTAA
G3MB1-b	TGGGTAGGGCGGGTTTAATTTTTTTTTAAACCC-Biotin
G4MB3-b	AGGG TTAGGGTTAGGGTTAGGGTTTAATTTTTTTTTAAACCCTAACCC-Biotin
G3MB1-F	TGGGTAGGGCGGGTTTAATTTTTTTTTAAACCC-Folate

All the DNA sequences can fold into hairpin structure by self-hybridizing of underlined fragments.

Figure S1. The sequence optimization of G3MB and G4MB. (A) In the presence of different G3MB. (B) In the presence of different G4MB. The concentrations of G3MB, G4MB and ThT were 100 nM, 100 nM and 6 μM. The insets respectively display the DNA sequences of G3MB0-4 and G4MB0-4. Error bars are standard deviation of three repetitive experiments.

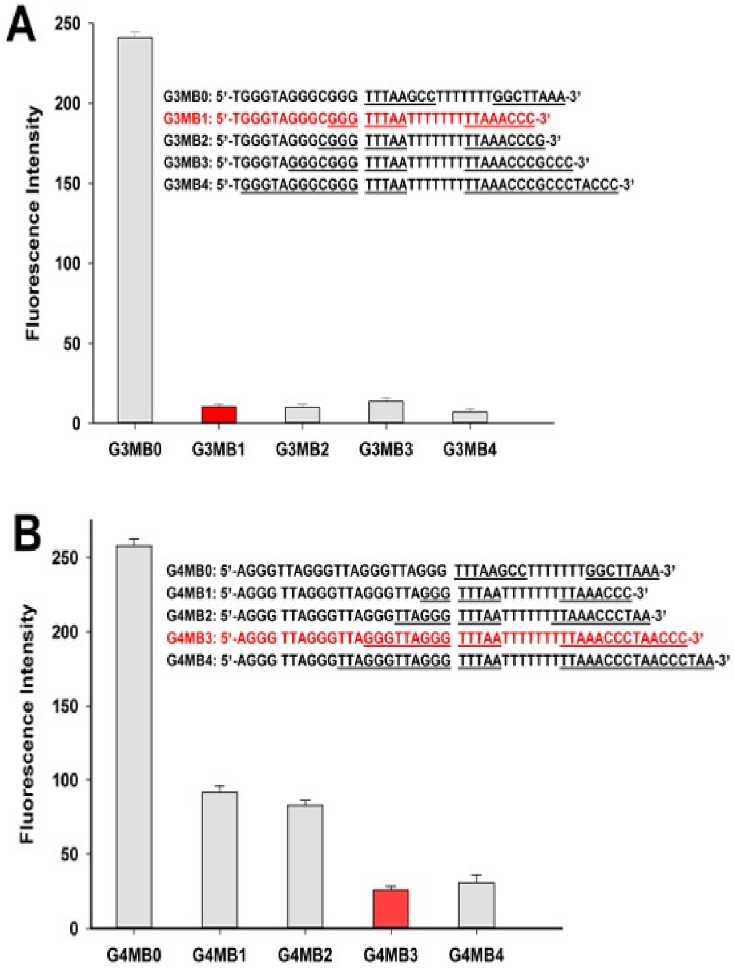

Figure S2. The optimization of Exo III concentrations and enzymolysis time. (A) Fluorescence intensity of ThT and G3MB1 or G4MB3 at different Exo III concentrations, incubated for 2 h at 37 °C in dark. (B) Fluorescence intensity at different enzymolysis time, incubated at 37 °C in dark in 0.5 U Exo III; [G3MB1] = 100 nM; [G4MB3] = 100 nM; [ThT] = 6 μM; $[Mg^{2+}]$ = 10 mM; $[K^{+}]$ = 25 mM.

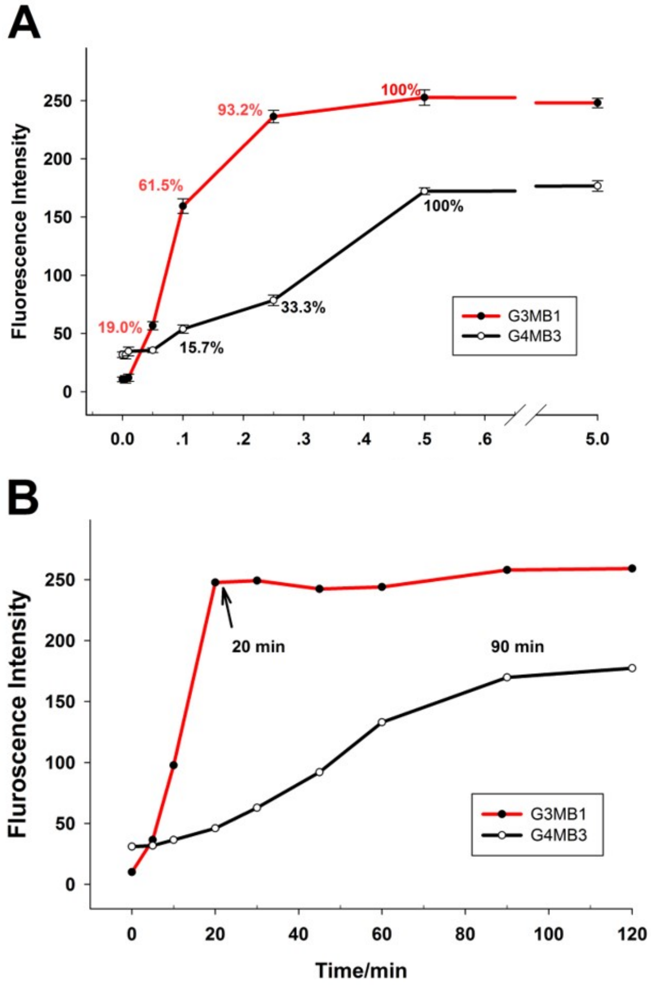

Figure S3. The SA detection by the G4MB3 biosensor. (A) Fluorescence spectra of the solution contained G4MB3-b, ThT and Exo III after incubation with varying concentrations of SA (0, 0.1, 0.5, 1, 5, 10, 25, 50, 75, 100, 200 ng) (B) Fluorescence intensity of the solution contained G4MB3-b, ThT and Exo III after incubation with varying concentrations of SA. Linear relation between the fluorescence intensity and the concentration of SA. [G4MB3-b] = 100 nM; [ThT] = 6 μM; [Mg^{2+}] = 10 mM; [K^+] = 25 mM; Exo III = 0.5 U.

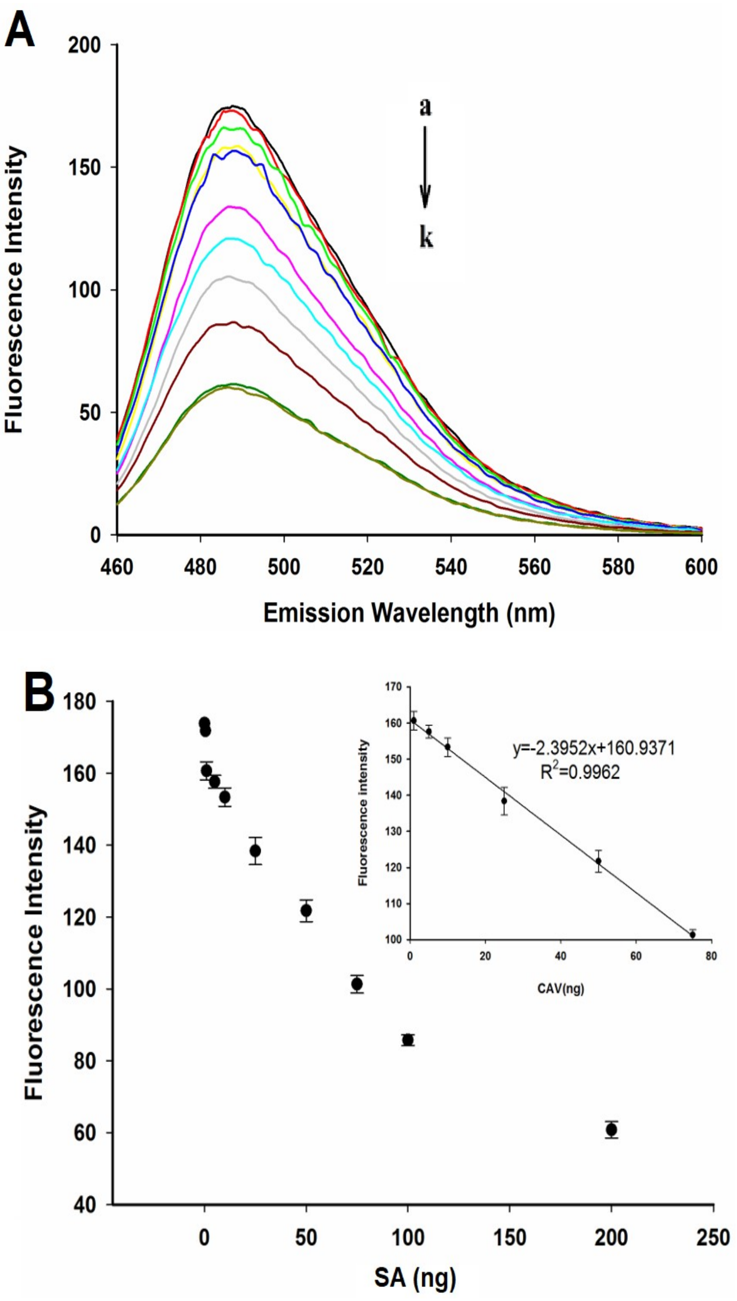

Supplement: Supplementary file 1 [file molecules-26-02962-s001.zip › molecules-1204618-supplementary.pdf]
